# Intra-fractional dosimetric analysis of image-guided intracavitary brachytherapy of cervical cancer

**DOI:** 10.1186/s13014-021-01870-x

**Published:** 2021-08-04

**Authors:** Junfang Yan, Jiawei Zhu, Kai Chen, Lang Yu, Fuquan Zhang

**Affiliations:** 1 Department of Radiation Oncology, Peking Union Medical College Hospital, Chinese Academe of Medical Sciences & Peking Union Medical College, Beijing, 100730 China; 2 Department of Radiation Oncology, Sun Yat-Sen University Cancer Center, State Key Laboratory of Oncology in South China, Collaborative Innovation Center for Cancer Medicine, 651 Dongfeng Road East, Guangzhou, 510060 Guangdong China

**Keywords:** Cone beam computed tomography, Image-guided intracavitary brachytherapy, Intra-fractional variation, Cervical cancer

## Abstract

**Background:**

To assess the intra-fractional dosimetric variations of image-guided brachytherapy of cervical cancer.

**Methods:**

A total of 38 fractions (9 patients) undergoing brachytherapy for cervical cancer underwent a CT scanning for treatment planning (planning CT) and a Cone-beam CT (CBCT) scanning immediately prior to delivery (pre-delivery CBCT). The variations of volumes as well as the dosimetric impact from treatment planning to delivery (intra-application) were evaluated. The dose volume histogram parameters including volume, D90 of high-risk clinical target volume (HRCTV) and D2cc of organs at risk (OARs) were recorded.

**Results:**

The relative differences (mean ± 1SD) of the volume and D90 HRCTV across the two scans were − 2.0 ± 3.3% and − 1.2 ± 4.5%, respectively. The variations of D2cc for bladder, rectum, sigmoid and small intestine are − 0.6 ± 17.1%, 9.3 ± 14.6%, 7.2% ± 20.5% and 1.5 ± 12.6%, respectively. Most of them are statistically nonsignificant except the D2cc for rectum, which showed a significant increase (*P* = 0.001). Using 5% and 10% uncertainty of physical dose for HRCTV at a 6 Gy × 5 high-dose-rate schedule, the possibility of total equivalent doses in 2 Gy fractions (EQD2) lower than 85 Gy is close to 0% and 3%, respectively. Performing similar simulation at 15% and 20% uncertainty of a 4 Gy physical dose for OARs, the possibility of total EQD2 dose exceeding 75 Gy is about 70%. Less than 1% of the total EQD2 of OARs would exceed 80 Gy.

**Conclusions:**

Average intra-fractional dosimetric variation of HRCTV was small in an interval of less than 1 h, and the possibility of total EQD2 exceeding 85 Gy is higher than 97%. The intra-fractional dosimetric variations of OARs might result in an overdose for OARs in a single fraction or the whole treatment. It is necessary to detect unfavorable anatomical changes by re-imaging and take interventions to minimize applied doses and reduce the risk of complications.

**Supplementary Information:**

The online version contains supplementary material available at 10.1186/s13014-021-01870-x.

## Introduction

Radiotherapy is an essential component of curative treatment of locally advanced cervical cancer, which includes external beam radiotherapy (EBRT) and brachytherapy (BT) [1]. Based on detailed information of the anatomical situation and applicator position, three-dimensional image-guided brachytherapy (3D-IGBT) achieves the precise planning with improved disease control, overall survival (OS) and complications over conventional brachytherapy [2–5]. Although MRI-based 3D-IGBT is the gold standard for cervical cancer [6], CT-based planning provides useful information for discrimination of the organs at risk (OARs), which has a similar effect as MRI [7]. In addition, CT-based 3D-IGBT is easier to achieve due to the popularity of CT simulators in most medical centers [7–10]. Using the pre-brachytherapy MRI as a reference to assess tumor extension, the contouring of CT-based 3D-IGBT can be more precise and be used as an alternative when MRI-based IGBT is difficult to achieve [7, 11].

However, the effect of intra-fractional variation remains an issue in 3D-IGBT for cervical cancer [12]. These intra-fractional target and OARs variations might result from changes in location relative to the applicator, variations of shape and/or filling status of OARs, and patient transfer. Several studies have reported the intra-fractional dosimetric variations in the 3D-IGBT for cervical cancer, which might require repetitive imaging and predelivery intervention treatment [12–15].

Two common approaches used in the reported studies to calculate the applied dose are based on repetitive scanning with CT imaging [12, 15], and MRI imaging [13, 14, 16]. In most trials, patients are usually transferred from the brachytherapy suite to CT/MRI unit for repetitive scanning. Cone-beam CT (CBCT) is a popular imaging method that provides valuable 3D information of the patient for treatment verification and plan of the day selection [17, 18]. Although CBCT exhibits a lower soft tissue contrast than CT, the main advantage of using CBCT is the capability of performing pre-delivery scan without transferring the patient from the CT unit to the brachytherapy suite. Limiting the patient’s motion is expected to limit postinsertion applicator motion, which in return provides real-time anatomical information and leads to more accurate calculation of the applied dose. In previous studies, CBCT with/without ultrasound was successfully utilized for 3D planning in high-dose-rate (HDR) BT for cervical cancer [19, 20]. Recent studies tried to calculate the applied dose based on CT-to-CBCT using deformable image registration algorithms (DIR) or directly calculation [21–23].

As far as we know, this was the first study to analyze and quantify intra-fractional variation of the high-risk clinical target volume (HRCTV) and OARs based on planning CT and pre-delivery CBCT for brachytherapy. Pre-delivery CBCT data are used to investigate variations in the applied dose distributions as compared to the dose distribution on planning CT. The HRCTV and OARs were contoured on the pre-delivery CBCT that were taken before each treatment, and the applied dose to these organs was assessed based on the patient’s real-time anatomy.

## Method

### Patients

A total of 9 patients were selected for the study according to the following criteria: (A) The pelvic MRI scan was completed at the time of diagnosis and the first-fraction of brachytherapy. (B) No previous history of abdominal and pelvic surgery. The cancer was classified as FIGO Stage IB1 for 2 patients, Stage IIA for 2 patients, Stage IIB for 4 patients, and Stage IIIB for 1 patient.

### Treatments

EBRT was delivered by intensity modulated radiation therapy to the whole pelvis, and a dose of 50.4 Gy in 28 fractions was delivered over a period of 5½ weeks. All the patients received five sessions of high-dose-rate ^192^Ir brachytherapy, which was usually administered twice per week. Consequently, 38 fractions of brachytherapy were available for this study. In principle, the prescribed dose was 6 Gy at HRCTV in each brachytherapy session. A separate insertion was used for each fraction with the Fletcher/Utrecht CT/MR applicator (Elekta, Stockholm, Sweden). The applicator was inserted into the vagina, and wet gauze was then packed on its anterior and posterior sides. During the insertion, the patients were placed on a bed board, and a fixation device was used to fix the applicator firmly to the bed board to reduce the movement of the applicator during patient transfer. A Foleys catheter was inserted for continuous drainage, ensuring an empty bladder. All patients were asked to defecate before insertion and no rectal tubes were inserted.

The CT image for treatment planning (the pre-delivery CT) was obtained at 2.5 mm thickness after the insertion of the applicator, using the Brilliance CT Big Bore (Philips Healthcare, Best, the Netherlands). The HRCTV and OARs (bladder, rectum, sigmoid, and small intestine) were then contoured according to Groupe Européen de Curiethérapie and European Society for Radiotherapy Oncology (GEC-ESTRO) recommendations using MRI as a reference cognitively [24, 25]. The treatment plan for IGBT based on the pre-delivery CT was then created; this was then approved by an experienced medical physicist and a radiation oncologist. The treatment planning system used for the brachytherapy was Oncentra version 4.1 software (Elekta, Stockholm, Sweden), and the dose was optimized by using inverse planning simulated annealing algorithm and manual optimization. After the completion of the CT-based treatment planning, patients were transferred to the brachytherapy suite and moved to the Trilogy linear accelerator (Varian Medical Systems, California, USA). Immediately after obtaining pre-delivery CBCT image at 2.5 mm thickness, the dose was delivered in situ.

### Dose and DVH evaluation of planning CT and pre-delivery CBCT

Volume, the D90 HRCTV, and D2cc of the OARs were calculated [26]. The brachytherapy dose was converted into the equivalent doses in 2 Gy fractions (EQD2) using the linear model with α/β = 10 Gy for HRCTV and α/β = 3 Gy for OARs [27]. The dose constraints were D2cc < 90 Gy for the bladder, D2cc < 75 Gy for the rectum, sigmoid and small intestine.

Re-calculation of the dose to the HRCTV and OARs at pre-delivery CBCT was performed. The planning CT and pre-delivery CBCT images were imported into the Eclipse version 8.6 software (Varian Medical Systems, Palo Alto, CA, USA), and both images were rigidly fused by matching the inserted applicator as a fiducial marker. The original contours of planning CT were re-sampled on pre-delivery CBCT. The contours were adapted according to the anatomy visible on the pre-delivery CBCT. The planned dose was mapped onto the pre-delivery CBCT; the planning CT dose placed on the pre-delivery CBCT was regarded as the applied dose. A single experienced observer reviewed all contours to reduce inter-observer contouring variation. D90 HRCTV and D2cc OARs values of the adapted contours were determined and converted to EQD2. Structure volumes were also calculated on the basis of the contouring of the rectum, bladder, sigmoid and small intestine. Percentage differences in structure volumes and dose-volume histogram (DVH) parameters were calculated using the following formula:

$${\mathrm{Difference}}\,(\% ) = \frac{{{\mathrm{Value}}_{{{\mathrm{pre}}}} - {\mathrm{Value}}_{{{\mathrm{plan}}}} }}{{{\mathrm{Value}}_{{{\mathrm{plan}}}} }} \times 100$$

We analyzed the mean and variability of the dosimetric difference and DVH value calculated for all cases, for each patient and for each fraction, and we also analyzed the trends. In order to evaluate the clinical importance of the observed dosimetric uncertainties, typical clinical scenarios for an HDR BT treatment were simulated to assess the effect of uncertainties on total accumulated dose. 4000 simulations were run according to a N (di, σ^2^) distribution, where di was the physical fractional dose and σ was the standard deviation (SD) of intra-application variations for target or OARs [14], as shown in the Additional file 1: Table S1.

Relevant σ levels were chosen according to the uncertainty level of D90 HRCTV and D2cc OARs in our study, as shown in Table 1: 5% and 10% for D90 HRCTV, and 15% and 20% for OARs according to the observed levels. In each group simulation, five uncertainties were produced randomly for five HDR brachytherapy. Each simulated fractional dose was transformed into biologically equivalent dose in 2 Gy fractions (EQD2), and thereafter all fractions were added and summed up with the EBRT EQD2 dose in order to obtain total EBRT + BT EQD2 dose. Results of the 4,000 simulations were evaluated and compared with the expected dose.

**Table 1:** Dosimetric variations for the HRCTV, bladder, rectum, sigmoid and intestine between planning CT and pre-delivery CBCT

	CT	CBCT	Variation	Range of difference	*P*
*HRCTV*					
Volume (cc)	54.1 ± 17.2	53.1 ± 16.9	− 2.0 ± 3.3%	0.02–6.8	< 0.001
D90 (cGy)	606.4 ± 39.3	599.1 ± 45.8	1.2 ± 4.5%	0.1–79.1	0.120
D90 (EQD2, cGy)	813.0 ± 72.6	800.1 ± 83.6	1.2 ± 6.1%	0.1–156.0	0.139
Bladder					
Volume (cc)	81.4 ± 53.5	81.7 ± 48.0	7.9 ± 36.7%	0.3–146.1	0.957
D2cc (cGy)	391.6 ± 74.8	389.2 ± 71.9	− 0.6 ± 17.1%	1.8–167.8	0.810
D2cc (EQD2, cGy)	552.5 ± 151.1	542.6 ± 151.2	2.7 ± 26.6%	4.2–355.2	0.772
*Rectum*					
Volume (cc)	46.2 ± 13.9	41.9 ± 15.5	− 6.9% ± 34.1%	0.02–22.5	0.001*
D2cc (cGy)	302.9 ± 84.4	327.0 ± 86.9	9.3 ± 14.6%	0.6–168.0	0.001*
D2cc (EQD2, cGy)	379.1 ± 157.1	425.7 ± 169.3	15.0 ± 24.0%	0.7–336.2	0.001*
*Sigmoid*					
Volume (cc)	24.8 ± 15.72	24.28 ± 11.1	19.9 ± 68.2%	0.2–48.9	0.763
D2cc (cGy)	301.5 ± 85.8	312.5 ± 74.6	7.2 ± 20.5%	1.7–130.3	0.127
D2cc (EQD2, cGy)	376.9 ± 146.6	393.7 ± 130.4	11.4 ± 31.9%	2.8–210.4	0.192
*Small intestine*					
Volume (cc)	401.0 ± 115.0	394.0 ± 145.7	− 0.5 ± 26.7%	2.3–360.7	0.705
D2cc (cGy)	411.2 ± 60.3	418.2 ± 83.4	1.5 ± 12.6%	0.8–138.8	0.422
D2cc (EQD2, cGy)	591.9 ± 130.9	614.2 ± 190.0	3.4 ± 20.7%	2.0–353.6	0.293

Relative differences between parameters (volume, D90 and D2cc) from two images sets were calculated as Difference = (Valuepre − Valueplan)/Valueplan. A positive value means that the volume/dose obtained for pre-delivery image was higher than that on the plan image

*P < 0.05

### Statistical analysis

A total of 76 image series were obtained for patients enrolled in this group, including 38 planning CT image series and 38 pre-delivery CBCT image series as shown in Additional file 1: Table S2. DVH parameters of HRCTV and OARs were collected and tested for normality. If they were in accordance with the normality test, the t-test was used for analysis. Wilcoxon signed rank sum test was performed if they were non-normally distributed. All the tests were two-sided, with *P* < 0.05 considered statistically significant. Statistical analysis was performed using SPSS 23.0 (IBM Corp, NY, USA).

## Result

The relative systematic and random variations of HRCTV and OARs between planning CT and pre-delivery CBCT are reported as mean (± 1SD uncertainties) in percentage in Table 1. Note that a positive difference indicates that the pre-delivery CBCT value is greater than the planning CT value. The mean time interval between the planning CT and pre-delivery CBCT acquisition was 53 min (range 39–92 min).

## Dosimetric variation for HRCTV

**Fig. 1:**
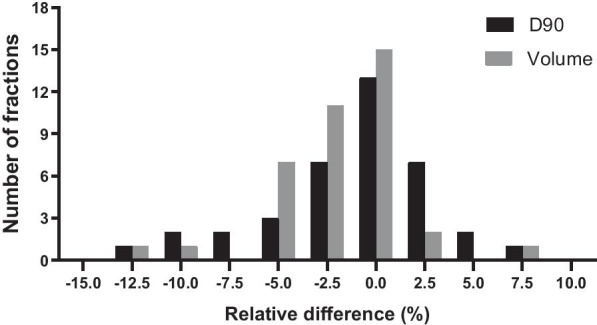
The histogram of the number of fractions for relative difference in the volume and D90 of HRCTV

The mean (± 1SD) variations between the planning CT and pre-delivery CBCT images were − 2.0 ± 3.3% for the HRCTV volume (*P* < 0.001) and 1.2% ± 4.5% for D90 physical dose (*P* > 0.05). The proportion of D90 dose difference within ± 5% is 76.3% (29/38), and the proportion exceeding ± 10% is only 7.9% (3/38) with a maximum change of − 12.7% (Fig. 1). The mean (± 1SD) variation of D90 (EQD2) is 1.2 ± 6.1% (*P* > 0.05), and the maximum value of absolute change is 1.6 Gy. The proportion of changes within ± 0.5 Gy was 73.7% (28/38), and the proportion of changes exceeding ± 1 Gy was 10.5% (4/38).

## Dosimetric variation for OARs

The mean values and variation of the structure volume and DVH parameter between the planning CT and pre-delivery CBCT for OARs are presented in Table 1. The mean (± 1SD) changes in structure volume, D2cc physical dose and EQD2 was − 6.9 ± 34.1%, 9.3 ± 14.6% and 11.3 ± 31.9% for the rectum, which was statistically different (*P* < 0.05). No statistically significant differences were found in the variations of structure volumes, D2cc physical dose and EQD2 for bladder, sigmoid and small intestine (all *P* > 0.05). The mean value in the DVH parameters of bladder and small intestine were within ± 3% for the D2cc physical dose and ± 5% for the EQD2 dose. However, large variations up to 1.7 Gy was observed for the D2cc physical dose of bladder.

**Fig. 2:**
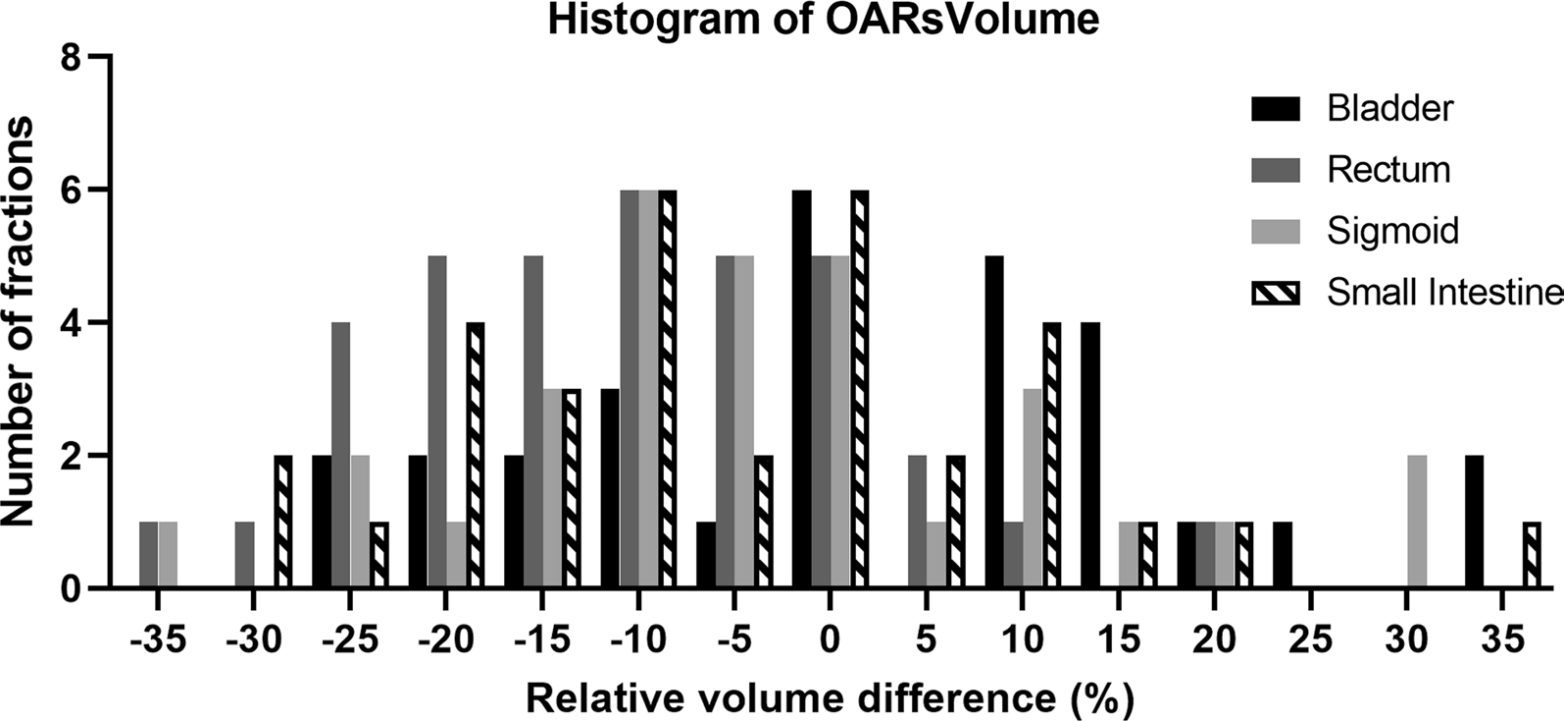
The histogram of the number of fractions for relative volume difference of OARs

**Fig. 3:**
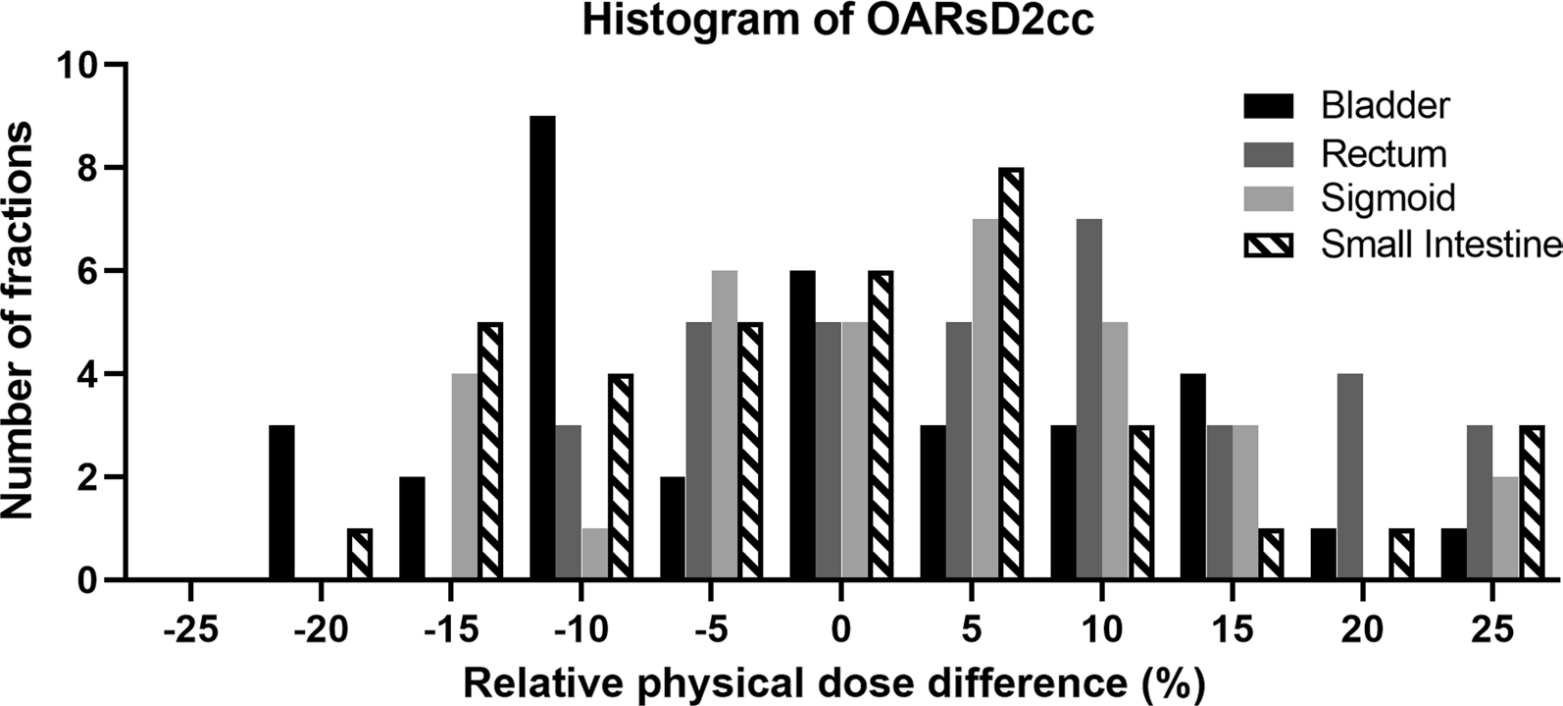
The histogram of the number of fractions for relative physical dose difference in the D2cc of OARs

Figures 2 and 3 present the histogram of the number of fractions for relative difference in the DVH parameters for OARs. The average volume variation and uncertainty of sigmoid tended to be higher than other OARs. The relative volume differences exceeding 20% were found for 34.2% of the fractions in the sigmoid. For most of the fractions, the relative physical dose differences were within 0% ± 15%; however, the relative dose differences exceeding 20% were found for 15.8% of the fractions in the bladder, 21.1% of those in the rectum, 18.4% of those in the sigmoid, and 10.5% of those in the small intestine. The maximum physical dosimetric variation was 37.6% for the bladder, 28.1% for the rectum, 77.0% for sigmoid and 33.2% for the small intestine.

## Probability distribution of the total dose based on dosimetric uncertainty

### HRCTV

Results of the simulation of total treatment dose in EQD2, taking into account the dosimetric uncertainties of individual BT fractions are shown in Table 2. For a random uncertainty of 5%, the mean (± 1SD) total simulated dose is 89.6 ± 1.2 Gy. There were 31.7% probability that total simulated dose less than 89 Gy, and 2% probability that total simulated dose less than 87 Gy. The probability that the total dose is less than 85 Gy is close to 0%. A random uncertainty of 10% lead to random uncertainties (± 1SD) of the total simulated dose of 89.7 ± 2.5 Gy. The probability that the total dose is less than 89 Gy is 40%. There were 15% probability that total simulated dose was less than 87 Gy, and 3% probability that total simulated dose was less than 85 Gy.

**Table 2:** Dosimetric simulation of the D90 HRCTV (EQD2) at different uncertainty level

Uncertainty	5%	10%
EQD2(Gy)	89.6 ± 1.2	89.7 ± 2.5
< 90 Gy	63.0%	55.6%
< 89 Gy	31.7%	38.8%
< 88 Gy	10.2%	25.1%
< 87 Gy	1.9%	13.5%
< 86 Gy	0.2%	6.1%
< 85 Gy	0.03%	2.8%
< 80 Gy	0	0

The di is 6 Gy for each fraction, and the σ^2^ are 0.3 Gy and 0.6 Gy when the uncertainty are 5% and 10%, respectively. And the total EQD2 is the sum of the BT dose and EBRT dose (49.56 Gy, 50.4 Gy/28f, α/β = 10)

### OARs

In this study, the average D2cc dose of OARs was about 3.5 Gy. The uncertainty in a single fraction of D2cc physical dose for OARs was about 15% and 20%. The similar simulation process was carried out for the OARs taking into account the dosimetric uncertainties, as shown in Table 3. The probability of EQD2 exceeding 75 Gy was about 6% and 14% in rectum, sigmoid and small intestine, respectively. Less than 1% of the total EQD2 of OARs would exceed 80 Gy.

**Table 3:** Dosimetric simulation of the OARs D2cc (EQD2) at different uncertainty level

Dose level	3.5 Gy		4 Gy	
Uncertainty	15%	20%	15%	20%
EQD2(Gy)	71.3 ± 2.4	71.6 ± 3.1	76.7 ± 3.0	77.0 ± 4.0
> 75 Gy	5.8%	13.7%	71.3%	68.0%
> 76 Gy	2.7%	8.2%	58.5%	58.9%
> 77 Gy	1.0%	4.7%	45.2%	49.4%
> 78 Gy	0.4%	2.5%	32.3%	39.4%
> 79 Gy	0.1%	1.2%	21.5%	30.1%
> 80 Gy	0	0.6%	12.6%	22.5%
> 85 Gy	0	0	0.3%	3.2%
> 90 Gy	0	0	0	0.1%

The di is 3.5 Gy or 4 Gy for each fraction, while the uncertainty level is 15% or 20%. And the total EQD2 is the sum of the BT dose and EBRT dose (48.38 Gy, 50.4 Gy/28f, α/β = 3)

In fact, the average physical dose of D2cc in bladder, rectum and small intestine was about 4 Gy. Therefore, the simulation process was repeated again at an average dose of 4 Gy, as shown in the Table 3. Under the uncertainties of 15% and 20%, the probability of the intestinal D2cc EQD2 exceeding 75 Gy is about 70%, and the probability of exceeding the 80 Gy is about 10% and 20%, respectively.

## Discussion

In terms of HRCTV, the average volume depicted on CBCT was smaller than that depicted on CT. However, the absolute difference is only about 1 cc, which has little effect on the dosimetric variation. This could be attributed to that the applicator and the HRCTV were usually fixed together resulting in a constant relative position between them. Besides, the time interval between two image scans was short in this study. Our patient could start treatment in situ immediately after the pre-delivery CBCT scanning, which provided the real-time anatomical information and reduced the variations caused by patient transfer. Such in-room imaging for evaluating intra-fractional variation haven’t been reported. Considering the accuracy and contouring error of different planning systems, this significant difference mainly affects D98 HRCTV, while the impact on D90 HRCTV can be ignored [28]. The systematic and random variation of D90 HRCTV in our study was 1.2 ± 4.5%, which was similar to previous studies [13, 24], as shown in Table 4.

**Table 4:** Summary of studies on intra-fractional variation of OARs

	Nesvacil 2013 [14]	Miyasaka 2020 [12]	Yan 2021	Nomden 2014 [13]	Simha 2014 [15]
Image	MRI & CT	CT	CT & CBCT	MRI	MRI & CT
Fraction	4F	6 Gy	6 Gy × 5F	7 Gy × 4F	7 Gy × 4F
Interval	3–5 h	43 min	53 min	3.9 h (2.8–5.5)	2 h (0.5–3.5)
D90 HRCTV	− 2.5% ± 10.8%	–	− 1.2 ± 4.5%	− 0.1 ± 0.5 Gy	–
D2cc Bladder	1.3% ± 17.7%	2.4 ± 8.8%	− 0.6 ± 17.1%	0.1 ± 1.1 Gy	0.5 ± 0.4 Gy
D2cc Rectum	3.8% ± 20.5%	− 2.3 ± 9.9%	9.3 ± 14.6%	0.4 ± 1.5 Gy	0.3 ± 0.3 Gy
D2cc Sigmoid	− 2.3% ± 23.5%	–	7.15 ± 20.5%	0.4 ± 1.2 Gy	0.6 ± 0.6 Gy

The results of Nesvacil et al., Miyasaka et al. and Yan et al. were given in % of physical dose. The results of Nomden et al. were given in physical dose, while the results of Nomden et al. were given in EQD2. Yan et al. represent the results of our study

The average D2cc change of the bladder was only − 0.6%, which was lower than 9.3% of the rectum. In addition, the average difference of bladder volume between planning and pre-delivery images was only 0.30 cc with no significant difference (*P* > 0.05). Similarly, urinary catheters were used in all cases and resulted in a lower dosimetric variation in the bladder than the rectum in the study of Andersen et al. [29]. Without using urinary catheters, Miyasaka et al. found a greater D2cc variation for the bladder than that of the rectum due to the accumulation of urine [12]. In this study, all patients were catheterized for continuous drainage to reduce the variations of bladder volume and D2cc [30, 31]. However, this was contradicted with the recommendation that maintaining the standardized bladder volume can reduce the variation of bladder dose [32]. In addition, the full bladder pushed the intestine upward and thus protects the small intestine since the constraint dose for the intestine was lower than the that for bladder [9, 33]. Therefore, there might be two useful methods for clinicians to control the volume and dosimetric variation of bladder. The first method was to use urinary catheters for continuous drainage. Another method was to inject the same amount of saline into the bladder using catheters before planning CT scan and dose delivery [12].

Unlike the bladder, the rectum expands and contracts in the anterior-to-posterior direction due to gas movement. Much of the variation in the rectum was in the direction of dose reduction [12, 29]. In our study, the volume of rectum decreased from planning CT to pre-delivery CBCT while the D2cc increased. We found that the rectum on pre-delivery CBCT was closer to HRCTV in the anterior-to-posterior direction, although no quantitative analysis on the movement of rectum was performed. It could be assumed that a larger space left by the empty bladder allowed the rectum to be closer to HRCTV after planning CT scanning. Measures such as pre-delivery verification and rectal catheters could be used to avoid excessive rectal dose for long interval between treatment planning and delivery. Nomdem et al. found that rectal catheters help to reduce rectal dose, and even steps to control gas in the rectum might effectively prevent accidental changes in organs [13].

The uncertainty (1SD) of the physical dose of D2cc for bladder, rectum, sigmoid and intestine was 17.1%, 14.6%, 20.5% and 12.6%, respectively. As shown in Table 4, Nesvacil et al. found a similar uncertainty level for OARs as our study [14]. The sigmoid colon had the highest average dose uncertainty of 20%, which was consistent with its high activity. However, the uncertainty levels were only 8.8% and 9.9% for bladder and rectum in the study of Miyasaka et al. [12]. The longer interval between image acquisition and treatment delivery, and the lower soft tissue contrast of CBCT and CT than MRI might be related with the larger uncertainty. However, the different treatment protocols, image techniques, segmentation standards and patient transfer modes made it difficult to compare the results from different studies.

Our simulations of delivered dose ranges for an example of a treatment with 6 Gy × 5 HDR BT evaluate the possibility of reaching the prescribed dose. In terms of D90 HRCTV, a dose of ≥ 85 Gy results in a local control rate of > 93% in intermediate size targets (HRCTV 20–30cm^3^) and > 86% in large targets (HRCTV 30–70cm^3^) at brachytherapy [24]. Another study by Domopoulos et al. [34] on the relationship between DVH parameters and local control rate found that the local control rate can reach > 95% at a D90 HRCTV dose of ≥ 87 Gy. In this study, at the uncertainty level of 10% in single fraction, the probability of total D90 EQD2 < 87 Gy is about 15%, and 3% for D90 EQD2 < 85 Gy. There is a risk that the received dose is lower than the recommended dose at an uncertainty of 10%. When increasing deliver dose to 6.2 Gy each fraction, the probability of total EQD2 of < 87 Gy and < 85 Gy is about 3% and 0.3% at an uncertainty level of 10%, respectively. It should be noted that this result is only applicable to 5 fractions HDR BT and a 50.4 Gy/28f external irradiation. In the study of Nesvacil et al. [14], an uncertainty of 10% and a total EQD2 of 90 Gy would result in a dose uncertainties of 3.3 Gy in the 7 Gy × 4 HDR BT. In our study, the corresponding dose uncertainty is 2.5 Gy in the 6 Gy × 5 HDR BT at an uncertainty of 10% and a total EQD2 of 90 Gy.

Controlling the D2cc EQD2 dose of bladder, rectum, sigmoid colon and small intestine lower than 90 Gy, 75 Gy, 75 Gy and 75 Gy can reduce the incidence of complications [24]. Our simulation results suggest that the probability of exceeding the constraint dose is less than 0.1% for EQD2 of bladder due to its high radiation tolerance. The sigmoid had the highest average D2cc uncertainty of 20%, which is consistent with the high mobility of sigmoid colon [35]. When the average applied dose is 4 Gy, the possibility of exceeding 75 Gy is 68% for EQD2 of the sigmoid. In the absence of definite data regarding the sigmoid toxicity and the high uncertainty [24], there is no need to compromise the HRCTV dose to keep the sigmoid dose within limits [15]. Although the uncertainty of rectum is lower than that of sigmoid (15% vs 20%), there might be a large variation in the total EQD2 dose of the rectum and recto-sigmoid junction due to the high average dose. Pre-delivery re-scanning helps to identify unfavorable movement and volumes change of bladder and rectum [12, 15]. What’s more, active preliminary treatment such as inserting catheter was recommended to control bladder volume or rectal gas to reduce intra-fractional variations.

There were some limitations to this study. First, this is a single-institution study with a small sample size that included only 9 participants (38 HDR fractions). Whether patient characteristics and treatment plans would cause dosimetric variation could not be valued. For example, none of the patients were catheterized for the rectum and the same fraction schedule was used in this study. Further studies with larger sample sizes from multiple institutions are needed to compare the difference between different preparations and treatment plans. Second, different imaging techniques were used for treatment planning and pre-delivery rescanning in order to limit the motion of patients and shorten the time interval. Uniform image technique could be used to reduce the intra-observer variation, and the patients would be able to received radiation in situ in such modality. Third, CBCT images include larger amounts of scattering and poorer resolution than CT, making it difficult for drawing organ wall contours and calculating the maximal dose precisely [36]. Although our result showed the possibility of CBCT for contouring and direct dose calculation, specialized calibrations and optimizations to reduce scattering should be considered in further studies. Last but not least, the use of planning CT and pre-delivery CBCT could not completely cover the entire intra-fraction motion. The variation of OARs during the minutes of treatment were missing in this sense.

## Conclusion

In conclusion, our results quantified the volume and dose changes caused by intra-fractional variation. The volume and relative position to applicator of HRCTV was stable in a short time interval leading to a small intra-fractional dosimetric variation. Most variations of the D90 EQD2 are within a clinical acceptable level and the possibility of total EQD2 exceeding 85 Gy is higher than 97%. Due to the greater changes of position and volume, the intra-fractional dosimetric variation of OARs was higher than that of HRCTV. This might result in an overdose in a single fraction or the constraint of individual OAR for the whole treatment (includes EBRT). Our finding supported the necessity to detect unfavorable anatomical changes by re-imaging prior to dose delivery. Interventions should be taken into consideration to minimize delivered doses and reduce the risks of complications, especially when the planning DVH parameters of rectum and small intestine were close to the limits.

## Supplementary Information

**Additional file 1.** **Table S1.** The dosimetric simulation process of bladder. The di is 4 Gy for each fraction, and the σ^2^ is 0.6 Gy when the uncertainty is 15%. The simulation formula is NORMINV (RAND (), 4, 0.6) using Excel (Microsoft Corporation). The EQD2 of brachytherapy (BT) is the sum of the 5 fraction. And the total EQD2 is the sum of the BT EQD2 and EBRT EQD2 (48.4 Gy, 50.4 Gy/28f, α/β = 3). Part of the data is shown in the following table. **Table S2.** Demographic features of the 9 patients. **Figure S1.** The correlation between time interval and dosimetric variation of bladder. The time interval between two image scans in this study ranged from 39 to 92 minutes, and the median time interval was 53 minutes. There was no obvious correlation between the variation of bladder D2cc and the length of time interval as shown in the following figure. There were similar results for the rectum, sigmoid colon, and small intestine.

## Data Availability

Owing to data privacy policy at our facility, publication of patient-related raw data is not possible.

## Abbreviations

HRCTV: High-risk clinical target volume
OARs: Organs at risk
EBRT: External beam radiotherapy
BT: Brachytherapy. 3D-IGBT: three-dimensional image-guided Brachytherapy
CBCT: Cone-beam computed tomography
EQD2: Equivalent doses in 2 Gy fractions
DVH: Dose-volume histogram
HDR: High-dose-rate
