# Supplementary material for: Intra-fractional dosimetric analysis of image-guided intracavitary brachytherapy of cervical cancer
Source: Radiat Oncol. 2021 Aug 4;16:144. doi: 10.1186/s13014-021-01870-x (PMC8335895; doi:10.1186/s13014-021-01870-x)

**Table S1.** The dosimetric simulation process of bladder. The di is 4 Gy for each fraction, and the σ^2^ is 0.6 Gy when the uncertainty is 15%. The simulation formula is NORMINV (RAND (), 4, 0.6) using Excel (Microsoft Corporation). The EQD2 of brachytherapy (BT) is the sum of the 5 fraction. And the total EQD2 is the sum of the BT EQD2 and EBRT EQD2 (48.4 Gy, 50.4 Gy/28f, α/β = 3). Part of the data is shown in the following table.

| Fraction 1 | Fraction 2 | Fraction 3 | Fraction 4 | Fraction 5 | BT EQD2 | Total EQD2 |
| --- | --- | --- | --- | --- | --- | --- |
| 3.3 | 4.0 | 4.2 | 4.1 | 4.5 | 28.4 | 76.8 |
| 2.9 | 3.4 | 3.6 | 4.0 | 3.3 | 22.4 | 70.8 |
| 3.7 | 3.2 | 4.2 | 3.6 | 3.8 | 25.0 | 73.4 |
| 3.5 | 2.1 | 3.2 | 3.4 | 3.7 | 20.0 | 68.3 |
| 2.8 | 3.8 | 4.6 | 3.1 | 3.0 | 22.9 | 71.3 |
| 3.7 | 3.9 | 3.3 | 3.6 | 3.5 | 23.7 | 72.1 |
| 3.7 | 2.9 | 3.7 | 4.5 | 3.7 | 24.9 | 73.3 |
| 4.1 | 3.3 | 3.7 | 4.1 | 2.9 | 24.3 | 72.7 |

**Table S2.** Demographic features of the 9 patients

| Patient No. | Age | FIGO stage | Pathologic type | Analyzed series |
| --- | --- | --- | --- | --- |
| 1 | 62 | IIB | Squamous | 5 |
| 2 | 56 | IIIB | Squamous | 5 |
| 3 | 51 | IIA2 | Squamous | 1 |
| 4 | 44 | IB1 | Squamous | 5 |
| 5 | 45 | IIB | Adeno | 5 |
| 6 | 54 | IIB | Squamous | 5 |
| 7 | 42 | IIB | Squamous | 5 |
| 8 | 50 | IIA1 | Squamous | 2 |
| 9 | 60 | IB1 | Squamous | 5 |

**Figure S1.** The correlation between time interval and dosimetric variation of bladder. The time interval between two image scans in this study ranged from 39 to 92 minutes, and the median time interval was 53 minutes. There was no obvious correlation between the variation of bladder D2cc and the length of time interval as shown in the following figure. There were similar results for the rectum, sigmoid colon, and small intestine.


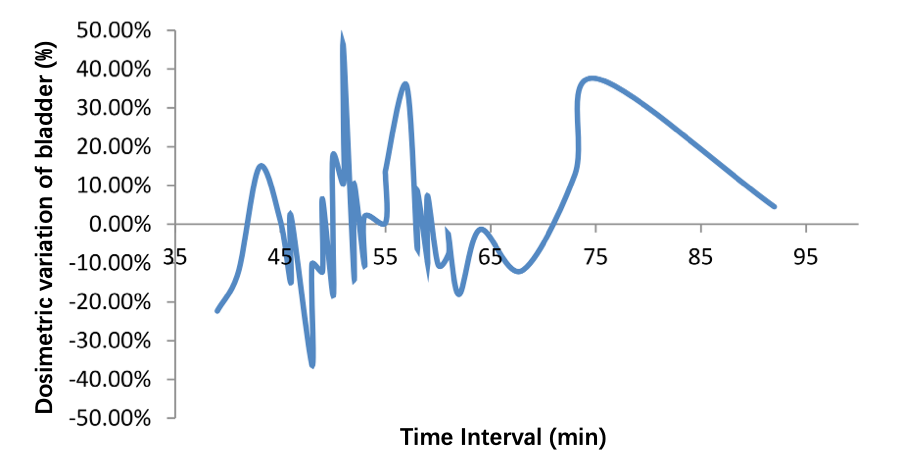

Supplement: Supplementary file 1 — Additional file 1. Table S1. The dosimetric simulation process of bladder. The di is 4 Gy for each fraction, and the σ2 is 0.6 Gy when the uncertainty is 15%. The simulation formula is NORMINV (RAND (), 4, 0.6) using Excel (Microsoft Corporation). The EQD2 of brachytherapy (BT) is the sum of the 5 fraction. And the total EQD2 is the sum of the BT EQD2 and EBRT EQD2 (48.4 Gy, 50.4 Gy/28f, α/β = 3). Part of the data is shown in the following table. Table S2. Demographic features of the 9 patients. Figure S1. The correlation between time interval and dosimetric variation of bladder. The time interval between two image scans in this study ranged from 39 to 92 minutes, and the median time interval was 53 minutes. There was no obvious correlation between the variation of bladder D2cc and the length of time interval as shown in the following figure. There were similar results for the rectum, sigmoid colon, and small intestine. [file 13014_2021_1870_MOESM1_ESM.docx]
